# Medical students’ perceptions and curriculum preferences for robotic surgery in Oman: a cross-sectional study

**DOI:** 10.1007/s11701-026-04008-x

**Published:** 2026-09-28

**Authors:** Abdullah Al-Lawati, Azzan Al-Wahshi, Lubna Al-Hashmi, Ali Abduwani, Ibrahim Al-Sibayi, Ahmed Al-Rawahi, Said Al-Busafi, Hani Al-Qadhi

**Affiliations:** 1 Department of Surgery, Sultan Qaboos University Hospital, Al-Khoudh 123, Muscat, Oman; 2 College of Medicine and Health Sciences, Sultan Qaboos University, Al- Khoudh 123, Muscat, Oman; 3 Department of Surgery, Khoula Hospital, Al Fahal St, Muscat, Oman; 4 Department of Medicine, College of Medicine and Health Sciences, Sultan Qaboos University, Al-Seeb, Muscat, Oman; 5 Medical Research Center, Sultan Qaboos University, Al Khoudh, Muscat, Oman; 6 Directorate General of Health Services, Dhofar, Oman

**Keywords:** Robotic surgery, Medical education, Educational needs assessment, Curriculum development, Simulation-based training, Surgical innovation, Attitudes, Oman, Minimally invasive surgery

## Abstract

Robotic surgery is an advanced form of minimally invasive surgery, and its use has continued to grow worldwide. Despite this, undergraduate medical education has not expanded at the same rate, and formal exposure to robotic surgery remains limited in many settings, including Oman. A structured educational needs assessment is an important first step in curriculum development. Understanding medical students’ perceived knowledge, attitudes, and preferred learning approaches can help guide the introduction of robotic surgery into undergraduate and postgraduate training. This study therefore aimed to assess medical students’ perceived knowledge, attitudes, and curriculum preferences regarding robotic surgery in medical training in Oman. This cross-sectional observational study used a validated questionnaire adapted from a published robotic-surgery study and distributed electronically to medical students at Sultan Qaboos University (SQU). It was used as a local educational needs-assessment tool to examine students’ perceived knowledge, attitudes, and preferred learning approaches regarding robotic surgery. Convenience sampling was used. Data were analyzed using IBM SPSS Statistics version 26.0, and *P* ≤ 0.05 was considered statistically significant. A total of 519 medical students were included in the study. Although 71.5% had heard of robotic surgery, 87.3% felt that their knowledge of the field was insufficient. Greater precision was identified most often as an advantage (67.2%), whereas higher cost was the most frequently reported disadvantage (82.3%). Some students remained hesitant about undergoing robotic surgery themselves. Despite this, support for incorporating robotic surgery into training was high, with 81.3% favouring its inclusion in the MD curriculum and 88.2% supporting its inclusion in residency training. Simulation-based teaching was the preferred learning method (73.5%), followed by attending robotic procedures during surgical rotations (66.5%). Most respondents also believed that Oman should invest in robotic surgery. Medical students in Oman generally had a positive view of robotic surgery, but self-perceived knowledge gaps and cost concerns remain. Robotic surgery education could therefore be introduced gradually, beginning with basic concepts, patient counselling, simulation-based learning, and supervised clinical exposure. These findings may help guide the curricular integration of this subject into undergraduate and postgraduate medical training in Oman.

## Introduction

Minimally invasive surgery (MIS) is a surgical philosophy in which primary operations are performed through small incisions [1]. The techniques it encompasses (including video endoscopy) have revolutionized surgery [2]. Under MIS, patients experience reduced postoperative pain (and hence reduced immobility-related morbidity), reduced surgical trauma and incision-related complications, earlier hospital discharge, shorter postoperative recovery, and improved cosmesis [3, 4].

Robotic surgery is among the most advanced forms of MIS, defined as surgical technology that places a computer-assisted electromechanical device between the surgeon and patient [5, 6]. The da Vinci Surgical System (dVSS) remains the dominant commercially available platform, in which a surgeon-controlled console remotely manipulates the robot’s arms in a master-slave configuration [1, 4, 7]; a full technical description is beyond the educational scope of this study.

In theory, robotic platforms address certain limitations of conventional laparoscopy, such as disrupted hand-eye coordination due to the two-dimensional view and the fulcrum effect, which requires counterintuitive hand movements [1, 2]. Furthermore, robotic surgery has shown a shallower learning curve than laparoscopy, whose technical difficulty has limited its widespread adoption in some specialties [7]. Nevertheless, the actual benefits of robotic surgery over conventional laparoscopy remain debated, with high costs and longer setup times representing clear disadvantages [1].

Almost all laparoscopic procedures have been attempted robotically [1], with an upward trend towards its use in urgent and emergency settings, including cholecystectomies, colectomies, and inguinal and ventral hernia repairs [8] but robotic surgery has found its main applications in urology and gynaecology [7]. By the end of 2023, 8,606 dVSS units were installed globally, 1,484 of which were in Asia, with approximately 2.29 million procedures performed that year [9].

In Oman, robotic surgery was introduced at the Royal Hospital in November 2025, initially in urology, with planned expansion to colorectal, obstetrics and gynecology, hepatobiliary and gastrointestinal, and cardiothoracic surgery [10]. Undergraduate medical students do not receive formal curricular exposure to robotic platforms. No published data describe how medical trainees or early-career clinicians in Oman view robotic surgery. This is important as the country continues to develop its healthcare services, since local evidence can help guide educational planning and resource allocation. Awareness, exposure, and acceptance of robotic surgery among medical trainees in Oman have not yet been formally studied. Understanding these views may help in planning how robotic surgery can be integrated into medical education and training. From an educational perspective, this can serve as a local educational needs assessment by identifying what students already know, what they feel they need to learn, and how they prefer to be taught. Kern’s six-step framework provides a useful basis for this approach, as it identifies learner needs first and then uses them to guide learning objectives, educational strategies, implementation, and evaluation [11]. Previous studies suggest that robotic surgery can be introduced during undergraduate medical training, although such teaching is still relatively uncommon. Simulation may be particularly useful in improving student engagement and preparedness [12, 13]. Against this background, the present study was conducted as an educational needs assessment to examine medical students’ knowledge, attitudes, and preferred methods of learning about robotic surgery, as well as its potential role in medical education in Oman.

## Materials and methods

### Study design

This cross-sectional observational study used a validated questionnaire as an educational needs assessment tool to evaluate the knowledge, attitudes, and learning preferences of Sultan Qaboos University (SQU) medical students regarding robotic surgery. The study used a non-probability convenience sampling technique. This study adhered to the Strengthening the Reporting of Observational Studies in Epidemiology (STROBE) guidelines for cross-sectional studies reporting.

### Conceptual framework

Kern’s six-step approach to curriculum development was used as the educational framework for interpreting the study and translating its findings into curriculum-development recommendations. The present study primarily addresses the first two stages of the framework: problem identification and general needs assessment, and targeted needs assessment of learners. The remaining stages, including formulation of learning objectives, selection of educational strategies, implementation, and evaluation, were used to contextualize the curricular implications derived from the study findings. For Steps 1–4, limited formal exposure and perceived knowledge gaps were linked to problem identification and the general needs assessment (Step 1); students’ knowledge, attitudes, career interests, and learning preferences to the targeted needs assessment (Step 2); the identified gaps to proposed objectives covering basic principles, safety, cost, and patient counselling (Step 3); and preferred teaching methods to the selection of educational strategies (Step 4).

### Study duration

The study included a 1-month pilot phase involving a cohort of 15 students. The students were asked to fill the questionnaire and give qualitative feedback. The research team then made minor adjustments based on the feedback. The 15 pilot participants were excluded from the final analyzed sample. The finalized questionnaire was used for a 3-month data collection period.

### Questionnaire and data collection

Participants included medical students enrolled at SQU. The questionnaire was adapted from a previously published robotic-surgery questionnaire, including a previously validated instrument [14, 15]. Minor modifications were made to suit the local medical-student context and study objectives. The adapted questionnaire was piloted for clarity but was not formally revalidated. The survey was distributed electronically via Google Forms in English. English is the language of instruction in the SQU medical program and clinical teaching, and pilot participants were asked to comment on item clarity and comprehension. Participants provided informed consent at the beginning of the survey. Data were collected and managed using Microsoft Excel 2018.

### Survey sections

1. **Demographic Information:** included gender, age, nationality, academic status, career interest (surgical or non-surgical), self-perceived technological proficiency, previous exposure to robotic surgery, and belief regarding its availability in Oman.
2. **Knowledge About Robotic Surgery:** included sources of information, understanding of basic principles, and perceived advantages of robotic surgery.
3. **Attitudes Toward Robotic Surgery:** included views on the adoption of robotic surgery, patient acceptance, expected outcomes, surgeon competence and professionalism, and its possible expansion in Oman. Participants were also asked how robotic surgery could be integrated into MD and residency training.

The survey consisted mainly of single- and multiple-choice questions with predefined response options.

### Statistical analysis

Data were analyzed using IBM SPSS Statistics version 26.0 (IBM Corp., Armonk, NY, USA). Descriptive statistics were used to summarize the study variables. Quantitative variables were reported as medians with interquartile ranges (IQR), while categorical variables were presented as frequencies and percentages. We assessed associations between participant characteristics and willingness to undergo robotic surgery using the chi-squared test. For this analysis, willingness was grouped into willing (might consider, willing in most cases, or completely willing) and unwilling (refused or hesitant). A two-sided P value ≤ 0.05 was considered statistically significant. Specialty preference (surgical, non-surgical, or undecided) was also compared with awareness, perceived knowledge, the original five-level willingness item, attitudes, and support for curricular integration using the Chi-squared test. This dichotomization was used only for the regression analysis; the original five-level responses were retained for descriptive reporting. A multivariable binary logistic regression model was used to assess whether self-perceived technological proficiency independently predicted willingness to undergo robotic surgery, adjusting for gender, academic stage, and future career preference. Adjusted odds ratios with 95% confidence intervals were reported.

### Inclusion/Exclusion criteria and sample size

The study targeted all medical students at SQU, including both preclinical and clinical phases. The minimum sample size was calculated using the single-population proportion formula, assuming a 95% confidence level and a 5% margin of error. This gave a required sample of 385 participants. Of the 780 medical students invited, 589 responded, giving a response rate of approximately 75.5%. Seventy responses were excluded: 45 because the questionnaire was incomplete, 21 because the reported age was implausible or below 18 years, and 4 because they were duplicate submissions. The final analysis included 519 students, which exceeded the minimum required sample size.

### Ethical approval and confidentiality

#### Ethical approval

was obtained from the Medical Research and Ethics Committee (MREC) at SQU (MREC#3519). Participation was voluntary, and all data were stored in an encrypted, password-protected folder accessible only to the principal investigator and co-investigators. Participants could withdraw at any stage without consequence, and confidentiality was strictly maintained throughout the research process. The study adhered to all the principles of the Declaration of Helsinki.

## Results

### Sociodemographic data

The study included 519 medical students, comprising 172 males (33.1%) and 347 females (66.9%). Most participants (94.4%) were Omani, while only 5.6% were non-Omani living in Oman. The median (interquartile range) age of the participants was 21 (3) years. Most of the students who participated in the study had a GPA of 3.00-3.49 (39.9%). When asked about future interests (surgical vs. non-surgical specialty), most students (48.6%) reported not yet having decided on a specialty. Table 1 shows the sociodemographic characteristics of the study participants.

**Table 1:** Sociodemographic characteristics of the study participants

		*n* = 519	%
Gender	Male	172	33.1
	Female	347	66.9
**Nationality**	Omani	490	94.4
	Non-Omani	29	5.6
**Median age (interquartile range)**	21 (3) years		
**GPA**	Less than 2.00	9	1.7
	2.00-2.49	18	3.5
	2.50–2.99	123	23.7
	3.00-3.49	207	39.9
	3.5-4.00	113	21.8
	Would rather not say	49	9.4
**Future field of interest**	Not decided yet	252	48.6
	Surgical specialty	138	26.6
	Non-surgical specialty	129	24.9
**Do you consider yourself a tech-savvy person?**	Yes	390	75.1
	No	129	24.9
Academic stage	Preclinical	434	83.6
	Clinical	85	16.4

### Awareness and perceived knowledge

**Fig. 1:**
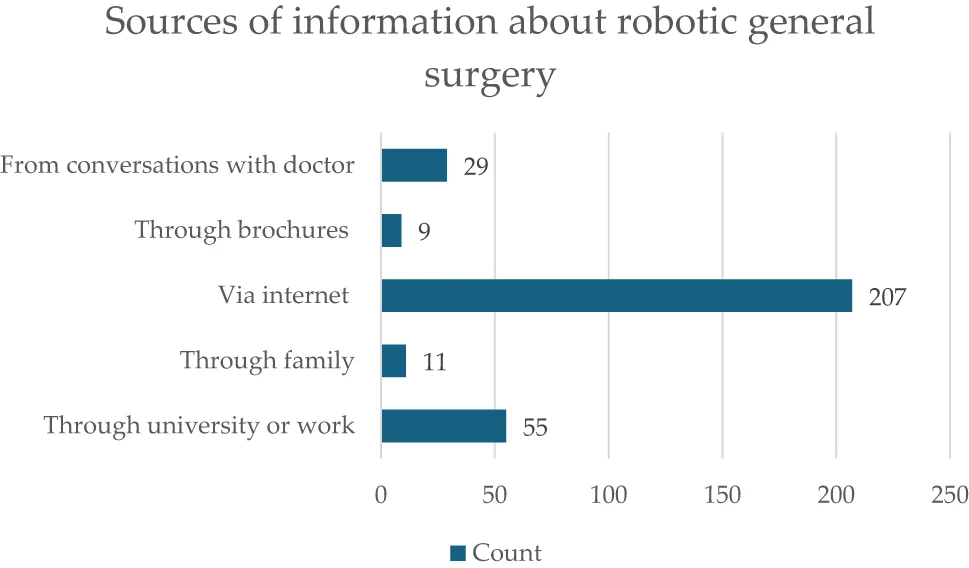
Sources of information about robotic general surgery among participants. Multiple responses permitted

Overall, 71.5% of the participants reported prior awareness of robotic surgeries. A small proportion, 14 participants (2.7%), indicated that they or a close family member had previously undergone a robotic surgical procedure. Approximately one-quarter of respondents (27.6%) believed that robotic surgery was available in Oman. Notably, most participants (87.3%) acknowledged insufficient knowledge of robotic surgery. Figure 1 shows sources of information about robotic general surgery among participants.

**Fig. 2:**
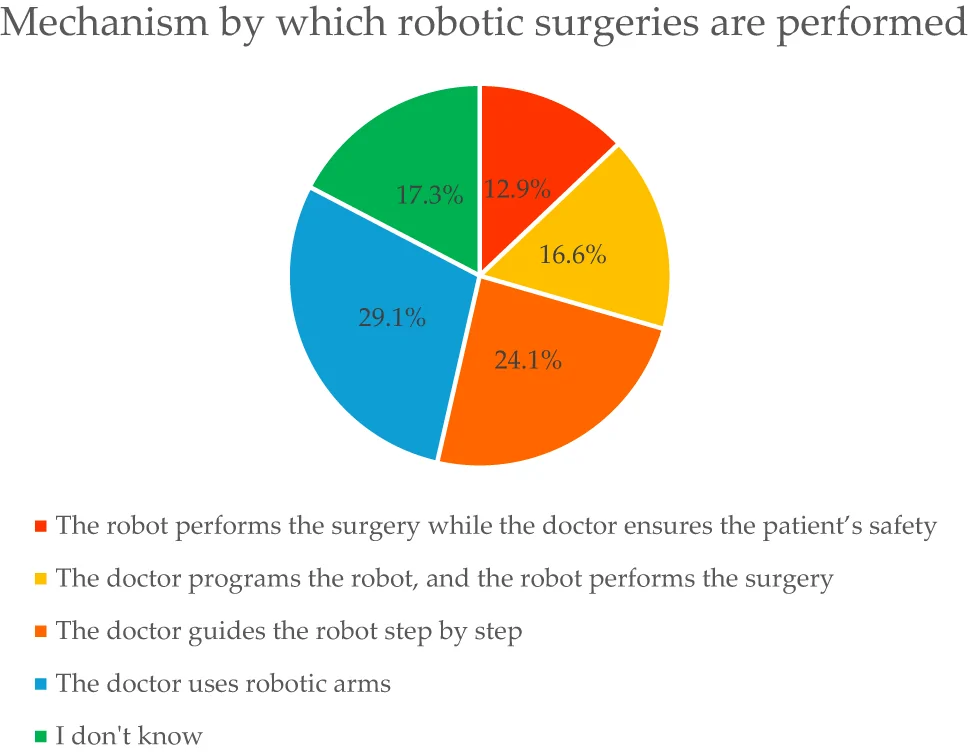
Participants’ perceptions regarding the mechanism by which robotic surgeries are performed

When asked about their understanding of how robotic surgeries function, the most common response was that the surgeon operates robotic arms to perform the procedure (29.1%). Figure 2 presents the distribution of participants’ perceptions regarding the mechanism of robotic surgery.

### Attitudes toward robotic surgery

When asked about the advantages of robotic surgery, the most commonly reported advantage was the belief that it offers greater precision than traditional surgery. In contrast, when asked about the disadvantages of robotic surgery, most participants reported that the higher cost compared with conventional surgery is the main disadvantage. Table 2 summarizes the advantages and disadvantages of robotic surgeries as perceived by the study population.

**Table 2:** Advantages and disadvantages of robotic surgeries as perceived by the participants

Robotic surgeries			
Advantage	*n* (%)	Disadvantage	*n* (%)
More precision in performing surgery	349 (67.2)	Higher cost compared to traditional surgery	427 (82.3)
Fewer surgical complications	142 (27.3)	Longer operation time	95 (18.3)
Shorter operating time and recovery	246 (47.4)	Difficulty in performing the procedure	135 (26)
Making the doctor’s work easier	224 (43.2)	No significant difference from traditional surgery	21 (4)

* Multiple responses permitted; percentages do not sum to 100.

When participants were asked about their willingness to undergo robotic surgery instead of traditional procedures, 17.7% of respondents reported that they would altogether refuse the idea. Meanwhile, 38.5% were hesitant to undergo robotic surgery, while 35.1% said they might consider it in certain situations. A smaller proportion (7.1%) were willing to undergo robotic surgery in most cases, and only 1.5% said they would be fully willing to choose it for any procedure. Table 3 shows the association between selected participant characteristics and willingness to undergo robotic surgery. A multivariable binary logistic regression was performed to identify independent predictors of willingness to undergo robotic surgery, adjusting for gender, academic stage, GPA, future career preference, and self-perceived technological proficiency. After adjustment, technological proficiency remained a significant independent predictor for willingness to undergo robotic surgery (OR = 1.62, 95% CI: 1.06–2.47, *p* = 0.025). Other factors including gender (OR = 0.93, 95% CI: 0.63–1.38, *p* = 0.716), academic stage (OR = 0.89, 95% CI: 0.54–1.46, *p* = 0.649), GPA (*p* = 0.199) and future career preference (*p* = 0.910) were not significant predictors of willingness to undergo robotic surgery.

**Table 3:** Association between participant characteristics and willingness to undergo robotic surgery

		Willing n (%)	Unwilling n (%)	p-value
**Gender**	**Male**	76 (44.2)	96 (55.8)	0.885
	**Female**	151 (43.5)	196 (56.5)	
**Self-perceived technological proficiency**	**Yes**	182 (46.7)	208 (53.3%)	0.019*
	**No**	45 (34.9)	84 (65.1)	
**Personal or family history of robotic surgery**	**Yes**	6 (42.9)	8 (57.1)	0.946
	**No**	221 (43.8)	284 (56.2)	
**Academic Stage**	**Preclinical**	188 (43.3)	246 (56.7)	0.663
	**Clinical**	39 (45.9)	46 (54.1)	

* Self-perceived technological proficiency: adjusted OR = 1.62, 95% CI: 1.06–2.47, *p* = 0.025, from multivariable logistic regression adjusting for gender, academic stage, GPA, and future career preference

Most participants agreed that Oman should invest in and expand robotic surgery (88.6%). When asked whether they believed robots could replace surgeons in the future, most participants said no (53.9%), while 39.7% said robots might somewhat replace surgeons. Only 6.4% believed that robots might completely replace surgeons in the future.

### Curriculum integration and learning preferences

**Fig. 3:**
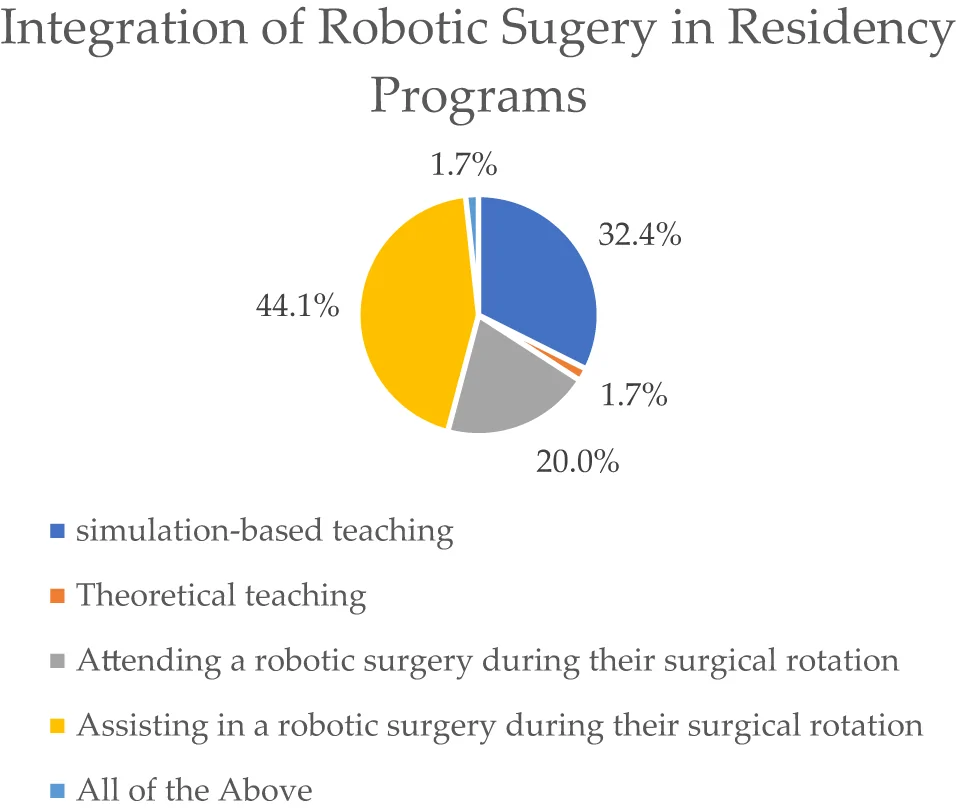
Participants’ suggestions on how robotic surgeries should be integrated into residency programs

Most students supported including robotic surgery in the MD curriculum (81.3%), while 18.7% did not. Simulation-based teaching was the most frequently preferred method (73.5%), followed by observing robotic procedures during surgical rotations (66.5%). Theoretical teaching alone was selected by 25.1% of students. Support for incorporating robotic surgery into residency training was similarly high (88.2%). Figure 3 summarizes the approaches students preferred for introducing robotic surgery into residency programs.

Table 4 summarizes attitudes toward integrating robotic surgery and future specialty preference. Support for including robotic surgery in residency training differed significantly by specialty preference (*p* = 0.010). Agreement was highest among students interested in surgical specialties (94.9%), compared with those interested in non-surgical specialties (88.4%) and those who were undecided (84.5%). Willingness to undergo robotic surgery also differed by specialty preference (*p* = 0.012); students with a stated surgical or non-surgical preference were more likely to report willingness for most or all procedures than undecided students. Support for MD-curriculum integration and investment in robotic surgery did not differ significantly between specialty groups.

**Table 4:** Attitudes toward robotic surgery and its integration into medical education, stratified by specialty preference (Not decided yet, Surgical, Non-Surgical)

Variable	Response	Not decided yet (*n* = 252)	Surgical specialty (*n* = 138)	Non-Surgical specialty (*n* = 129)	*p*-value
Interest in technology	Yes	190 (75.4%)	110 (79.7%)	90 (69.8%)	0.170
	No	62 (24.6%)	28 (20.3%)	39 (30.2%)	
Belief that robotic general surgery is available in Oman	Yes	74 (29.4%)	33 (23.9%)	36 (27.9%)	0.512
	No	178 (70.6%)	105 (76.1%)	93 (72.1%)	
Perceived sufficient information on robotic surgery	Yes	31 (12.3%)	22 (15.9%)	13 (10.1%)	0.343
	No	221 (87.7%)	116 (84.1%)	116 (89.9%)	
Would prefer robotics over traditional surgery	Absolutely not	42 (16.7%)	26 (18.8%)	24 (18.6%)	0.012
	I am not sure	102 (40.5%)	51 (37%)	47 (36.4%)	
	Yes, only for some surgeries	99 (39.3%)	43 (31.2%)	40 (31%)	
	Yes, for most surgeries	9 (3.6%)	14 (10.1%)	14 (10.9%)	
	Yes, for all surgeries	0	4 (2.9%)	4 (3.1%)	
Support for integrating robotic surgery into MD curriculum	Yes	203 (80.6%)	119 (86.2%)	100 (77.5%)	0.173
	No	49 (19.4%)	19 (13.8%)	29 (22.5%)	
Support for integrating robotic surgery into residency training	Yes	213 (84.5%)	131 (94.9%)	114 (88.4%)	0.010
	No	39 (15.5%)	7 (5.1%)	15 (11.6%)	
Belief that Oman should invest in/expand robotic surgery	Yes	219 (86.9%)	126 (91.3%)	115 (89.1%)	0.415
	No	33 (13.1%)	12 (8.7%)	14 (10.9%)	
Belief that robots could replace surgeons in the future	Yes	14 (5.6%)	9 (6.5%)	10 (7.8%)	0.865
	No	139 (55.2%)	76 (55.1%)	65 (50.4%)	
	Somewhat	99 (39.3%)	53 (38.4%)	54 (41.9%)	
Academic Stage	Preclinical	223 (88.5%)	111 (80.4%)	100 (77.5%)	0.012
	Clinical	29 (11.5%)	27 (19.6%)	29 (22.5%)	

### Awareness and attitude by academic stage

Awareness of robotic surgery differed significantly by academic stage, with a higher proportion of clinical students reporting prior awareness compared to preclinical students (87.1% vs. 68.4%, *p* = 0.001). Similarly, clinical students were more likely to perceive themselves as having sufficient knowledge about robotic surgery than preclinical students (24.7% vs. 10.4%, *p* < 0.001). Support for integrating robotic surgery into residency training was also significantly higher among clinical students than preclinical students (95.3% vs. 86.9%, *p* = 0.027). In contrast, support for incorporating robotic surgery into the MD curriculum did not differ significantly between the two groups (81.2% vs. 81.3%, *p* = 0.972).

## Discussion

In this study, we assessed medical students’ awareness, perceived knowledge, and attitudes toward robotic surgery in Oman, with particular attention to its possible role in medical education. As the use of robotic surgery continues to grow, understanding how future doctors view this technology is important, especially in countries such as Oman where its use is still developing. To our knowledge, this is the first study to examine medical students’ perceptions and curriculum preferences regarding robotic surgery in Oman. The main educational message is that students appear receptive to instruction in robotic surgery. Still, their self-reported knowledge gaps indicate a need for structured learning, beginning with the needs assessment and goal-setting steps of formal curriculum design before robotic surgery is introduced as a clinical service or training expectation.

A key finding was the contrast between relatively high awareness of robotic surgery and substantial self-perceived knowledge gaps. Although almost three-quarters of respondents reported having heard of robotic surgery, most said their knowledge of the technology was insufficient. Similar differences have been reported in regional literature. According to a Saudi Arabian study by Sultan et al., most medical students and surgical trainees were not only familiar with robotic surgery but also had a positive attitude toward it; 63.2% were positive, and 22.6% had prior knowledge or exposure during the course [15]. Similarly, Joiya et al., in a cross-sectional survey conducted in Pakistan, reported high awareness rates but identified knowledge gaps among medical and non-medical students, confirming that awareness does not always translate into an informed understanding of the aspects and applications of robotic surgery [16].

Participants identified fundamental features of robotic surgery, including the surgeon’s ability to control robotic arms and improve procedure accuracy. However, this understanding appeared largely theoretical and was accompanied by uncertainty, raising questions about the depth of theoretical familiarity without a practical basis. A recent systematic review and meta-analysis indicated that, although perceptions of medical students differed across countries, positive attitudes toward robotic-assisted surgery were often influenced by curriculum exposure and education [17].

In Oman, the main barrier appears to be limited exposure rather than resistance to robotic surgery. Although most respondents considered themselves technically capable, few believed that robotic surgery was available locally, and formal undergraduate exposure remains uncommon. This pattern suggests that the knowledge gaps identified in the study are more likely to reflect limited access to training and clinical experience than reluctance toward the technology. This is further supported by the strong interest in incorporating robotic surgery into both undergraduate and residency training. From a curriculum perspective, these findings can be viewed as an early needs assessment. Although students were generally aware of robotic surgery, most felt that their knowledge was limited and preferred simulator-based or clinical exposure over theoretical teaching alone. This gives the study clear educational value by showing what students already know, where they feel less prepared, and which teaching methods they prefer [12, 13].

This positive outlook was accompanied by a clear understanding of the surgeon’s central role, as most participants did not consider robotic systems substitutes for surgeons, but rather assistants that still rely on the surgeon’s experience and clinical judgement. Similar perceptions have been reported in regional and international research, suggesting that concerns about robotic systems replacing surgeons remain limited among medical trainees [17, 18]. However, the item on “making the doctor’s work easier” is ambiguous: robotic surgery may reduce physical strain while increasing cognitive demands related to situational awareness, team communication, and equipment troubleshooting.

In this study, students who were hesitant about personally undergoing robotic surgery appeared to be uncertain rather than opposed to the technology. Similar concerns have been reported in other settings, particularly around cost, access, and limited familiarity with robotic systems [16]. At the same time, robotic surgery has continued to expand across several specialties, especially in centres with greater institutional investment and established training pathways [19]. These willingness responses and beliefs about surgeon replacement were interpreted as separate attitudinal measures rather than indicators of educational readiness.

Participants expressed a strong preference for experiential learning approaches, favouring simulation-based and clinical exposure training over purely theoretical teaching. A practical curriculum in Oman could start with brief teaching on the indications, limitations, safety, cost, and patient counselling aspects of robotic surgery, followed by simulation and opportunities to observe robotic procedures during surgical rotations. At the residency level, training could then progress to patient safety, troubleshooting and emergency conversion, team communication, bedside-assistant roles, and, where resources permit, competency-based console training. This approach aligns with regional research suggesting that practical exposure is an important part of developing meaningful experience with robotic surgery [15, 20]. The findings highlight the value of experiential learning techniques for filling knowledge gaps, supporting informed acceptance, and guiding curriculum planning, rather than only measuring general acceptance of robotic surgery [12, 13]. Career preference was associated with willingness and support for residency integration, but not with support for MD integration or investment in robotic surgery. The broad support across groups suggests that basic robotic-surgery literacy was viewed as relevant beyond students already considering surgical careers.

Cost was the most commonly perceived drawback of robotic surgery, which is especially important in the context of Oman’s publicly funded healthcare sector, resource allocation, and cost-effectiveness. In contrast to private or mixed healthcare models, the development of robotic platforms in Oman needs careful justification in terms of clinical benefit, training opportunities, and sustainability. Cost-related barriers, including acquisition, maintenance, and per-procedure costs, have also been reported in implementation research [21, 22]. Physicians worldwide also cite cost as a key factor influencing adoption, even in the highest-income settings with institutional access to state-of-the-art technologies [21]. Student support for education should therefore not be interpreted as a mandate to acquire a platform immediately. Early curricular needs could be addressed through introductory teaching, shared simulation resources, and opportunities to observe robotic procedures at existing centres. Further clinical investment, however, should be guided by patient benefit, case volume, and cost-effectiveness.

Within Kern’s six-step framework for curriculum development [11], these findings fit mainly within the initial stages of problem identification and needs assessment. Although students were generally aware of robotic surgery, many felt that their knowledge was limited. They also preferred simulation and clinical exposure to didactic teaching alone. Subsequent curriculum development steps would include defining learning objectives, selecting appropriate educational strategies, planning for implementation, and establishing evaluation methods. This could be done through a staged undergraduate module that includes an introductory lecture, a simulator session, and observation of a robotic case, with knowledge and confidence assessed before and after the module. This would keep the focus on medical education rather than on the technology itself and give curriculum committees a practical starting point for introducing robotic surgery into training. More specifically, Step 1 is supported by the mismatch between awareness (71.5%) and insufficient perceived knowledge (87.3%), as well as low awareness of local availability (27.6%), defining the educational problem and general need. Step 2 is reflected in students’ self-perceived knowledge gaps, concern about cost (82.3%), strong support for including robotic surgery in the curriculum, and their preferred learning approaches. Together, these findings help define the target learners’ needs. For Step 3, they support learning objectives covering the basic principles, indications, limitations, safety, cost, and patient counselling related to robotic surgery. For Step 4, the students’ preference for simulation (73.5%) and clinical observation (66.5%) over theoretical teaching alone (25.1%) supports a staged approach with greater emphasis on practical exposure.

### Strengths and limitations

This study has notable strengths and certain limitations. Strengths include the large sample size, which exceeded the minimum required for adequate statistical power. In addition, using a questionnaire informed by published instruments provided a structured basis for assessing participants’ perceptions. As for limitations, the cross-sectional design limits our ability to establish causality, and reliance on self-reported responses introduces social desirability bias. In addition, this was a single-centre, student-based study, which may limit the generalisability of the results, as attitudes toward robotic surgery may depend on institutional exposure, access to resources, and local training culture. Although the response rate was 75.5%, non-responders may have been less interested in surgery, robotics, or technology, which could have led to overestimation of awareness and support for curricular integration.

The questionnaire was adapted from previously published instruments with minor modifications to suit the local context and study objectives. Although piloted for clarity, the adapted version was not formally revalidated, which may have affected its psychometric properties. Because the questionnaire focused on robotic general surgery, the findings should not be generalized to specialty-specific robotic practice without caution. Some response options in the item on how robotic surgery works may overlap conceptually, which may reduce its discriminant validity. The item on “making the doctor’s work easier” also did not distinguish physical from cognitive workload. Finally, the study did not objectively assess robotic surgery knowledge or evaluate a teaching intervention; therefore, the educational recommendations should be tested through prospective curriculum studies.

Future studies should also examine the views of practicing surgeons, consultants, and institutional decision-makers to provide a broader picture of readiness for robotic surgery in Oman.

### Future directions

Future studies should emphasize multicentre research that includes medical students from different institutions and training stages across Oman. In addition, future work should separately explore the perspectives of practicing surgeons and other stakeholders involved in implementing robotic surgery. Further analysis by intended specialty may help clarify whether students with different career plans place different value on robotic surgery. Future research should also test whether educational exposure changes what students know and how comfortable they feel with the technology. This could be examined using pre- and post-intervention studies of brief teaching sessions, simulator-based training, or supervised observation in the operating room, with outcomes including knowledge, confidence, clinical application, and readiness to counsel patients. In keeping with the evaluation stage of Kern’s framework, these studies should include objective educational measures as well as structured learner feedback. Comparing approaches such as simulation-based training and formal curricular modules could also help identify the most practical way to introduce robotic surgery into undergraduate and postgraduate medical education.

## Conclusions

Medical students in Oman were interested in robotic surgery, but many had limited knowledge of the field and little opportunity to gain practical exposure. Cost and access to training were the main concerns identified. These findings suggest a need for greater educational exposure, particularly as robotic surgery becomes more relevant to clinical practice. At present, this could be achieved through basic teaching, simulation, and observation of robotic procedures where available.

## Data Availability

The data are available upon request from the corresponding author.

## Appendix

### Appendix A. Study Questionnaire

**Consent Approval**.

1. Do you consent to participate in this study?

Response options: Yes; No (Please exit the survey).

Section 1. Sociodemographic Information and Personal Characteristics.

2. What is your gender?

Response options: Male; Female.

3. What best describes you?

Response options: Omani citizen; Non-Omani living in Oman.

4. What is your age? (answer with numerical value in English)

Response: ______.

5. What is your current cGPA? (responses are confidential)

Response options: Less than 2.00; 2.0-2.49; 2.5–2.99; 3.00-3.49; 3.50-4.00; Not applicable.

6. If you are a student, what is your future field of interest?

Response options: Not decided yet; Surgical specialty; Non-surgical specialty; N/A (not a student).

7. Do you consider yourself a tech-savvy person?

Response options: Yes; No.

Section 2. **Knowledge**.

8. Have you heard about robotic general surgery before?

Response options: Yes; No.

9. Have you or any of your close family members undergone a robotic general surgery procedure?

Response options: Yes; No.

10. Do you think robotic general surgery is available in Oman?

Response options: Yes; No.

11. Do you think you have enough information about robotic general surgery?

Response options: Yes; No.

12. If your answer was ‘Yes’, what is the source of your information about robotic general surgery?

Check all that apply.

Response options: From conversations with doctor; Through university or work; Through family; Via internet; Through brochures; I don’t know what robotic surgery is.

13. What do you think about how robots work in general surgery?

Response options: The robot performs the surgery while the doctor ensures the patient’s safety; The doctor programs the robot, and the robot performs the surgery; The doctor guides the robot step by step; The doctor uses robotic arms; I don’t know.

14. What are the advantages of robotic general surgery from your perspective?

Check all that apply.

Response options: More precision in performing surgery; Less surgical complications; Shorter operating time and recovery; Making the doctor’s work easier; I don’t know.

15. What are the disadvantages of robotic general surgery from your perspective?

Check all that apply.

Response options: Higher cost compared to traditional surgery; Longer operation time; Difficulty in performing the procedure; No significant difference from traditional surgery; I don’t know.

16. If given the option to undergo general surgery using a robot, would you prefer it over traditional surgery?

Response options: Absolutely not; I am not sure; Yes, only for some surgeries; Yes, for most surgeries; Yes, for all surgeries.

Section 3. **Implementation**.

17. Do you think robotic surgery should be integrated within the MD curriculum?

Response options: Yes; No.

18. If you answered ‘Yes’, how should medical students be introduced to robotics surgery?

Check all that apply.

Response options: Theoretical teaching; Practical sessions on a simulator; Attending a robotic surgery during their surgical rotation; I answered ‘No’ in the previous question; Other: __________.

19. Do you think robotic surgery should be integrated within the residency program?

Response options: Yes; No.

20. If you answered ‘Yes’, to what degree should residents be exposed to robotics surgery in your opinion?

Response options: Theoretical teaching; Practical sessions using a simulator; Attending a robotic surgery during their surgical rotation; Assisting in a robotic surgery during their surgical rotation; I answered ‘No’ in the previous question; Other: __________.

21. Do you think that Oman should invest in and expand robotic surgeries?

Response options: Yes; No.

22. Do you think using robots could replace surgeons in the future?

Response options: Yes; Somewhat; No.

23. Comments

Response: ________________________________________________________________.

## References

[1] Brunicardi FC, Andersen D, Billiar TR, Dunn DL, Kao LS, Hunter JG et al (2019) Schwartz’s Principles of Surgery, 11th edn. McGraw-Hill Education, New York

[2] Gharagozloo F, Patel VR, Giulianotti PC, Poston R, Gruessner RWG, Meyer M (2021) Robotic Surgery, 2nd edn. Springer, Cham

[3] Jaffray B (2005) Minimally invasive surgery. Arch Dis Child 90:537–542. 10.1136/adc.2004.062760 PMC1720379 15851444

[4] Diana M, Marescaux J (2015) Robotic surgery. Br J Surg 102(2):e15–e28. 10.1002/bjs.9711 25627128

[5] Kalan S, Chauhan S, Coelho RF, Orvieto MA, Camacho IR, Palmer KJ, Patel VR (2010) History of robotic surgery. J Robot Surg 4(3):141–147. 10.1007/s11701-010-0202-2 27638753

[6] Herron DM, Marohn M, SAGES-MIRA Robotic Surgery Consensus Group (2008) A consensus document on robotic surgery. Surg Endosc 22(2):313–325. 10.1007/s00464-007-9727-5 18163170

[7] Hussain A, Malik A, Halim MU, Ali AM (2014) The use of robotics in surgery: a review. Int J Clin Pract 68(11):1376–1382. 10.1111/ijcp.12492 25283250

[8] Lunardi N, Abou-Zamzam A, Florecki KL, Chidambaram S, Shih IF, Kent AJ, Joseph B, Byrne JP, Sakran JV (2024) Robotic technology in emergency general surgery cases in the era of minimally invasive surgery. JAMA Surg 159(5):493–499. 10.1001/jamasurg.2024.0016 PMC10918578 38446451

[9] Intuitive Surgical Inc. (2024) Annual report (Form 10-K) for the fiscal year ended December 31, 2023. U.S. Securities and Exchange Commission

[10] Ministry of Health, Sultanate of Oman. Royal Hospital performs Oman’s first robotic surgery, marking a major leap in medical innovation. 8 (2025) November https://moh.gov.om/en/media-center/health-achievements/royal-hospital-performs-oman-s-first-robotic-surgery-marking-a-major-leap-in-medical-innovation/

[11] Kern DE, Thomas PA, Hughes MT (eds) (2015) Curriculum Development for Medical Education: A Six-Step Approach, 3rd edn. Johns Hopkins University, Baltimore

[12] El-Mohamed J, Ahmadipour M, Warrier S, Costello AJ, Hii M, Mohan H (2025) Robotic surgical curriculum for medical students: a scoping review. J Robot Surg 19(1):496. 10.1007/s11701-025-02667-w PMC12364991 40830300

[13] Greenberg AL, Syed SM, Alseidi A, O’Sullivan PS, Chern H (2023) Robotic training for medical students: feasibility of a pilot simulation curriculum. J Robot Surg 17(3):1029–1038. 10.1007/s11701-022-01508-4 PMC10209258 36472723

[14] AlNaim AA, AlNaim N, Al Nasser F, Albash L, Almulhim M, Albinsaad L (2024) Public’s perception and knowledge of using robotics in general surgery in Eastern Region, Saudi Arabia. Ann Afr Med 23(3):299–306. 10.4103/aam.aam_182_23 PMC11364303 39034550

[15] Sultan I, Bardi MF, Baatta AM, Almaghrabi S, Mohammed RA (2022) Medical students’ attitude towards robotic surgery: a cross-sectional survey. J Med Educ Curric Dev 9:23821205211066483. 10.1177/23821205211066483 PMC8755928 35036565

[16] Joiya RS (2025) Awareness, perception, knowledge, and attitude toward robotic surgery amongst medical and non-medical population of Lahore. Br J Surg 112(Suppl 10). 10.1093/bjs/znaf128.469. znaf128.469

[17] Chand G, Singh S, Dhiraaj S, Kumar B, Shetty A, Halemani K, Ghatak T (2024) Perception of robotic-assisted surgery (RAS) among medical students: a systematic review and meta-analysis. J Robot Surg 18(1):95. 10.1007/s11701-024-01847-4 38413513

[18] Alhamaid YA, Alessa MY, Alhanfoush M et al (2025) Healthcare students’ attitude toward robotic surgery: a cross-sectional survey. Ann Afr Med 24(4):729–734. 10.4103/aam.aam_113_24 PMC12588411 40448442

[19] Sheetz KH, Claflin J, Dimick JB (2020) Trends in the adoption of robotic surgery for common surgical procedures. JAMA Netw Open 3(1):e1918911. 10.1001/jamanetworkopen.2019.18911 PMC6991252 31922557

[20] Barkati N, Ntefeh N, Okasha A et al (2023) Robotic assisted surgery in the United Arab Emirates: healthcare experts’ perceptions. J Robot Surg 17:2799–2806. 10.1007/s11701-023-01716-6 PMC10678779 37733210

[21] Lawrie L, Gillies K, Duncan E, Davies L, Beard D et al (2022) Barriers and enablers to the effective implementation of robotic assisted surgery. PLoS ONE 17(8):e0273696. 10.1371/journal.pone.0273696 PMC9423619 36037179

[22] Rosen RD, Edelman DA (2023) Should organizational investment in robotic surgical technology ever influence surgeons’ decisions about surgical approach to patients’ care? AMA J Ethics 25(8):E575–E582. 10.1001/amajethics.2023.575 37535501

